# Improved T1 fitting with the MOLLI sequence

**DOI:** 10.1186/1532-429X-15-S1-P50

**Published:** 2013-01-30

**Authors:** Mitchell A Cooper, Thanh Nguyen, Pascal Spincemaille, Jonathan D Kochav, Martin R Prince, Jonathan W Weinsaft, Yi Wang

**Affiliations:** 1 Radiology, Weill Cornell Medical College, New York, NY, USA; 2 BME, Cornell University, Ithaca, NY, USA; 3 Cardiology, Weill Cornell Medical College, New York, NY, USA; 4 School of Medicine, Duke University, Durham, NC, USA

## Background

T1 mapping has great potential for diagnosing diffuse cardiomyopathies. Modified Look Locker Inversion Recovery (MOLLI) is a promising 2D SSFP technique to measure T1 differences between healthy and diseased states in cardiac tissue. However, this method has been found to provide variable T1 values at different flip angles. Here we develop an improved fitting algorithm which accounts for the imperfect RF flip angle profile and SSFP signal evolution in MOLLI. We show that significantly improved T1 accuracy can be achieved across a larger range of flip angle in human calf muscle. This algorithm is then applied to MOLLI data obtained in the heart using an optimized flip angle.

## Methods

Imaging experiments were performed at 1.5T. For calf muscle (n=3), the MOLLI sequence was implemented with 16 inversion times (TI) = [50 150 250 350] (ms), TI+RR, TI+2*RR, TI + 3*RR, where RR = 750 ms is the R-R interval of the simulated cardiac gating (chosen intentionally to eliminate the effect of variable RR on the T1 accuracy). Three flip angles of 30°, 60° and 90° were used. MOLLI data fitting was performed using the original formula (T1 = T1* (B/A-1), where T1*, A and B are obtained by a three-parameter exponential fit) as well as the proposed algorithm. The gold standard inversion recovery spin echo (IR-SE) sequence was used to obtain reference T1 values.

Cardiac T1 mapping was then performed (n=5) using a 90° flip angle (determined to be optimal from the calf muscle experiment). 16 TI were acquired, however there was no free relaxation between Look Locker epochs in order to shorten scan time.

## Results

Table 1 compares in vivo calf muscle T1 values obtained with the original and proposed algorithms. While the original method provided highly variable T1's and larger residuals at higher flip angles (up to 14.9%), the proposed method provided accurate T1 and consistently low fitting residuals (1-2%) over all flip angles with minimal T1 error at 90°.

**Table 1:** 

Flip angle	T1 (ms)			Fitting residual (%)	
	
	*IR-SE*	*MOLLI*	*Proposed*	*MOLLI*	*Proposed*
30º		927	955	6.0	1.9
		
60º	980	969	969	12.1	1.1
		
90º		1032	978	14.9	1.1

**Figure 1:**
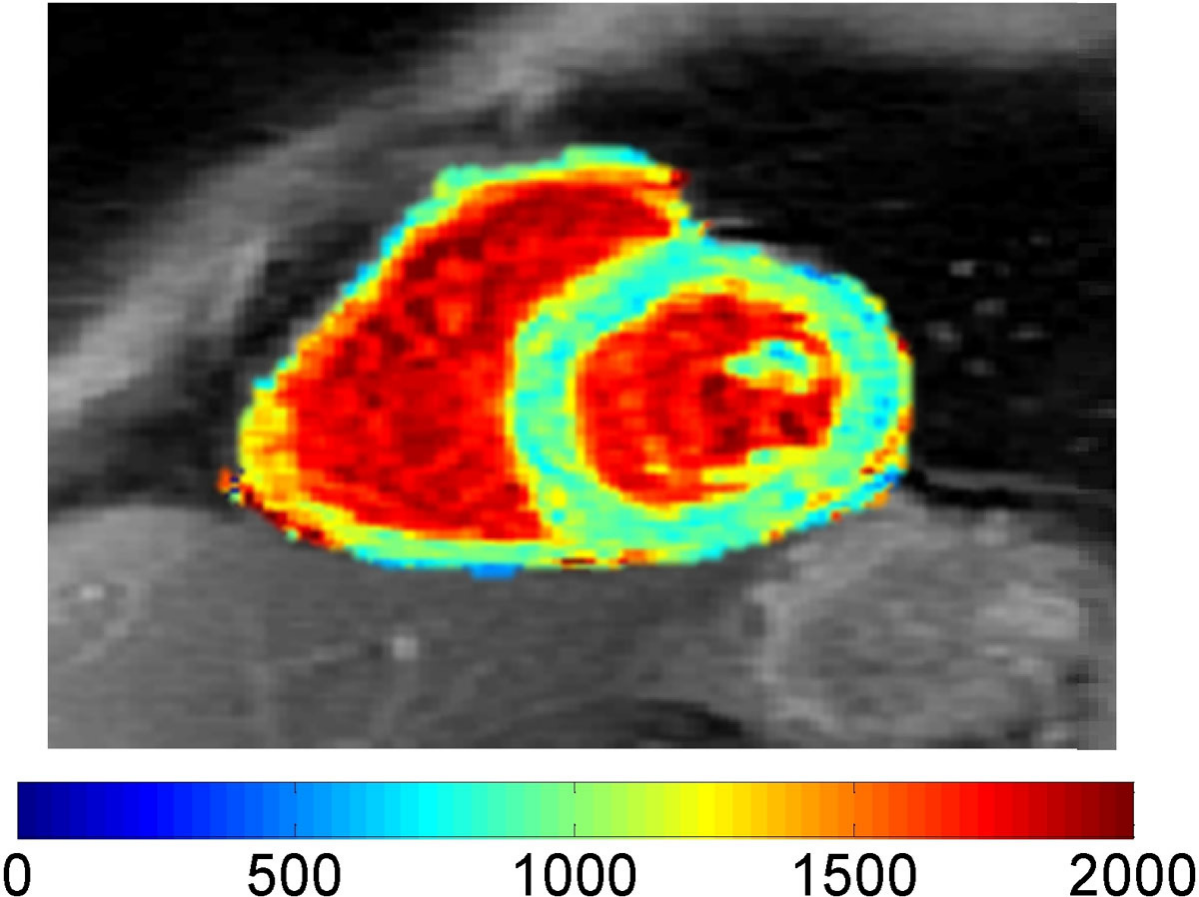


Figure 1 shows a representative cardiac short-axis T1 map for one volunteer. The myocardial T1 averaged over 5 volunteers was 986 ± 45 ms.

## Conclusions

While MOLLI allows for fast probing of tissue T1, the original fitting formula does not account for the effect of an imperfect RF excitation profile and only provides an approximation to the true SSFP signal evolution. Our improved fitting method can provide more accurate T1 results by incorporating these effects in the signal fitting.

## Funding

NSFGRFP; Grant number: DGE-0707428;

NIH; Grant number: HL064647

